# Supplementary material for: AtZAT4, a C2H2-Type Zinc Finger Transcription Factor from Arabidopsis thaliana, Is Involved in Pollen and Seed Development
Source: Plants (Basel). 2022 Jul 29;11(15):1974. doi: 10.3390/plants11151974 (PMC9370812; doi:10.3390/plants11151974)
Supplement: Supplementary file 1 [file plants-11-01974-s001.zip › plants-1789506-supplementary.pdf]

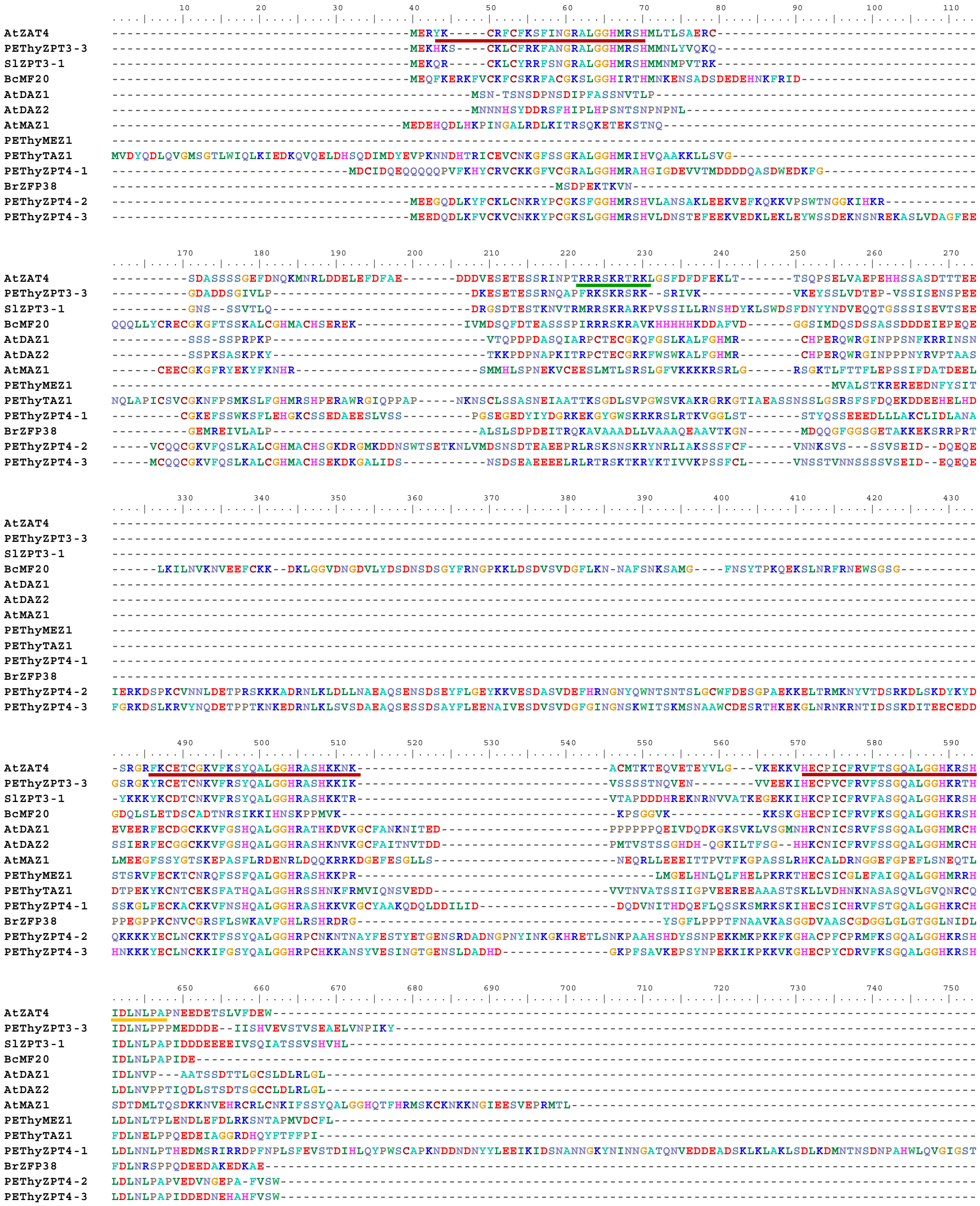

Figure S1. Multiple sequence alignment of deduced amino acid sequences of AtZAT4 and others C_2H_2 zinc-finger proteins (C_2H_2-ZFPs). The multiple alignment was done by using the MEGA X software (version 11.0) with ClustalW, and default parameters. Alignment editing was performed in BioEdit (version 7.2). Identification of conserved domains was performed in InterProScan, NLStradamus, and by previous literature. AtZAT4 C_2H_2-zinc finger domains, the Nuclear Localization Sequence, and the EAR motif are underlined in red, green, and yellow, respectively. For details, see the Materials and Methods section.

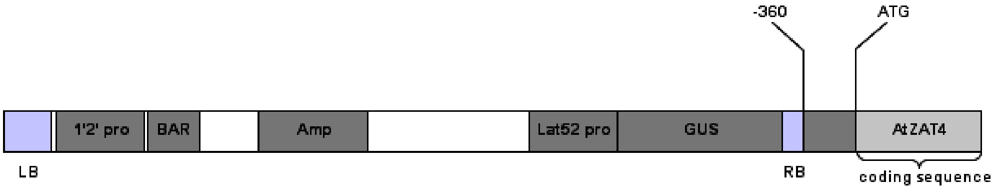

Figure S2. Proportional schematic representation of T-DNA insertion into the *Arabidopsis thaliana* genome and inside the *AtZAT4* promoter region. The T-DNA is the vector pCSA110, which carries the resistance to phosphinothricin gene (BAR; controlled by the 1'2' promoter), the ampicillin resistance gene (Amp), and the GUS marker gene (controlled by Lat52 promoter). RB and LB, right and left border of the T-DNA, respectively.

(A)

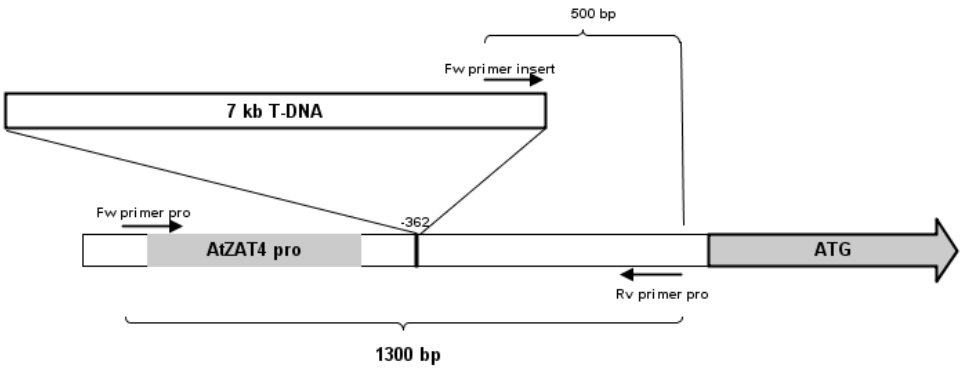

(B)

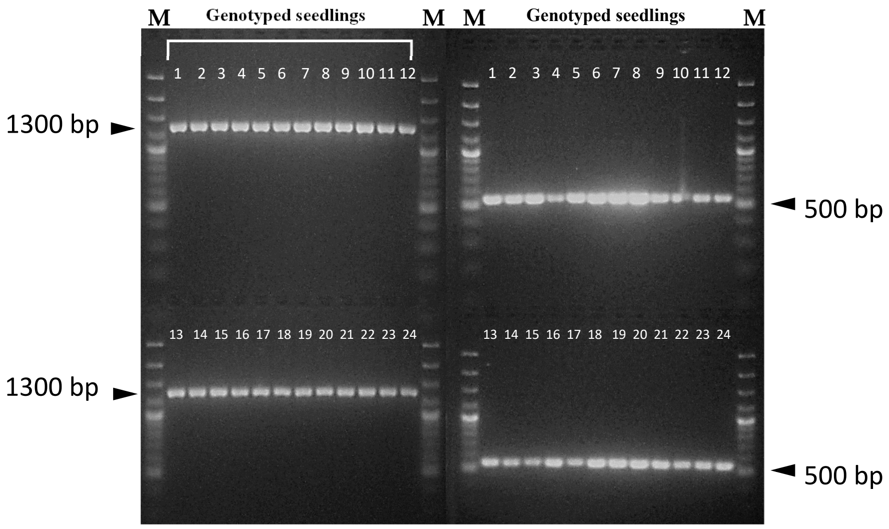

Figure S3. *Atzat4* (+/-) mutant genotyping from *Arabidopsis thaliana*. (**A**) Graphical representation of T-DNA insertion site and primers binding sites. Primer pro: primer promoter *AtZAT4* (pro*AtZAT4*), primer insert: primer T-DNA (pCSA110), see Table S1. (**B**) PCR products from 24 genotyped *A. thaliana* seedlings. The 1300 bp band represents the non-disrupted promoter and the 500 bp band indicated the T-DNA presence. M: 100 bp plus (Thermo Scientific, Waltham, MA, USA).

Table S1. Nucleotide sequence of primers used in RT-qPCR and in genotyping analyses.

Gene	Primer sequence (5′→3′)	Annealing temperature (°C)
AtZAT4	F: ACGGAAGTAACATCGGAGCAGGAA	60
	R: CCCGAATCACGCAAAGTCTGTTGT	60
AtF-box	F: TTTCGGCTGAGAGGTTCGAGT	60
	R: GATTCCAAGACGTAAAGCAGATCAA	60
pCSA110	F: ATTGATGAAACTGCTGCTGT	58
	R: GGTGATACATATCCAGCCAT	58
pro*AtZAT4*	F: GAGATCTAGAGGTTTGGCACCACCAT	57
	R: CGACGAGCTCGTACAGAACAGAGATA	57
